# Purinergic signalling in the cardiovascular system—a tribute to Geoffrey Burnstock

**DOI:** 10.1007/s11302-020-09734-x

**Published:** 2020-11-05

**Authors:** Vera Ralevic

**Affiliations:** School of Life Sciences, Queen’s Medical Centre, University of Nottingham, Nottingham, NG7 2UH UK

**Keywords:** ATP, Cardiovascular system, Purine receptors

## Abstract

Geoffrey Burnstock made groundbreaking discoveries on the physiological roles of purinergic receptors and led on P2 purinergic receptor classification. His knowledge, vision and leadership inspired and influenced the international scientific community. I had the privilege of spending over 10 years (from 1985) with Geoff at the Department of Anatomy and Developmental Biology, initially as a PhD student and then as a postdoctoral research fellow. I regarded him with enormous admiration and affection. This review on purinergic signalling in the cardiovascular system is a tribute to Geoff. It includes some personal recollections of Geoff.

## Classification of purine receptors

Geoffrey Burnstock is immortalized in purinergic receptor classification which he drove through his research and conceptualisation of the functional and molecular evidence. Geoff recognized the importance of research in the adenosine receptor field and functional differences compared with ATP/ADP to propose that there are two main families of purinergic receptors: P1 receptors for adenosine and P2 receptors which can be activated by purine nucleotides (ATP, ADP) [1]. Geoff further proposed that P2 receptors are divided into two functionally distinct classes: P2X and P2Y receptors [2]. P2 receptors are also activated by pyrimidine nucleotides and nucleotide sugars (UTP, UDP, UDP-glucose) [3]. Adenosine P1 receptors are divided into A1, A2A, A2B, and A3 subtypes which belong to the G protein-coupled receptor (GPCR) superfamily [4–6]. The 1990s were a vibrant time in Geoff’s laboratory as Geoff, together with Eric Barnard and colleagues, cloned the P2Y1 receptor [7] and news emerged about the cloning of the P2Y2 receptor [8] and first P2X receptors [9, 10], which contributed to the feeling of a gathering momentum in purinergic research. Adenosine receptors had already been cloned (see [4, 5]). Geoff’s excitement was palpable and infectious. Based on the molecular and functional evidence, Geoff proposed that P2 receptors are divided into two structurally and functionally distinct classes, ligand-gated ionotropic P2X receptors and G protein-coupled P2Y receptors [11].

There are seven subtypes of P2X receptors (P2X1–7) and these ligand-gated ion channels are activated by extracellular ATP. P2X receptors are permeable to cations leading to depolarization and Ca2+ influx through voltage-gated calcium channels or slower Ca2+-dependent responses [12, 13]. There are eight subtypes of G protein-coupled P2Y receptors: P2Y1, P2Y2, P2Y4, P2Y6, P2Y11, P2Y12, P2Y13, and P2Y14. P2Y receptors are activated by a variety of nucleotides including ATP, ADP, UTP, UDP, and UDP glucose [14–16]. P2Y1, P2Y2, P2Y4, and P2Y6 receptors couple to Gq proteins and activate phospholipase C (PLC), leading to an increase in inositol triphosphate (IP3) and intracellular Ca2+ levels and activation of protein kinase C (PKC); P2Y12, P2Y13, and P2Y14 receptors are coupled to Gi proteins and cause inhibition of adenylyl cyclase (AC) activity resulting in a reduction in intracellular cyclic adenosine monophosphate (cAMP); P2Y11 receptors couple to both Gs and Gq proteins to activate PLC and AC [15–18].

Purinergic receptors can undergo heteromeric complex formation between the families, e.g. P2Y1 and P2Y2 heteromeric association with A1 receptors [19, 20], within the families, e.g. P2Y1–P2Y12 complex formation [21], and also with other receptors, e.g. A1 receptors with β1 and β2 adrenoceptors [22]. P1 and P2 receptors are also functionally linked via the actions of cell surface ectonucleotidases which generate adenosine from the metabolism of nucleotides released from cells, permitting sequential activation of P2 and then P1 receptors. For example, in sympathetic cotransmission, ATP constricts blood vessels via smooth muscle P2X1 receptors, and its metabolite adenosine acts as a brake at prejunctional P1 (A1 subtype) receptors to inhibit neurotransmitter release [15]. Extracellular nucleotide levels are controlled through their hydrolysis by families of ectonucleotidases including the ectonucleoside triphosphate diphosphohydrolase (ENTPDase) family which rapidly hydrolyses extracellular tri - and diphosphate nucleotides (including ATP and ADP to AMP; UTP and UDP to UMP) and ecto -5’ -nucleotidase, the main enzyme responsible for the conversion of AMP to adenosine [23, 24], and also by their cellular reuptake via plasma membrane nucleoside transporters.

## P1 and P2 receptor expression in the cardiovascular system

P1 and P2 receptors are widely expressed throughout the cardiovascular system, and their effects include modulation of heart function, vascular tone, angiogenesis, and inflammation. P1 adenosine receptors expressed on the vascular smooth muscle, endothelium, and heart are generally vasodilator and cardioprotective [4, 5, 15, 16]. P2X and P2Y receptors are expressed in the heart and in general have ionotropic effects, amplify sympathetic neurotransmission, and increase myocyte contractility [15, 25–28]. The vascular smooth muscle expresses P2X1 receptors which mediate vasocontractile responses to ATP released as a co-transmitter with noradrenaline from sympathetic nerves. Pre-junctional purine receptors include A1 adenosine and P2Y receptors on sympathetic nerves and P2X2/3 receptors at the central terminals and axons of sensory nerves [15]. Vasocontractile P2Y2, P2Y4, and P2Y6 receptors, and in some vessels P2Y14 receptors, are also expressed in vascular smooth muscle, and vasorelaxant P2Y1, P2Y2, P2Y4, and P2Y6 receptors are expressed on the vascular endothelium [15, 16, 29–34]. Endothelial cells also express P2X4 receptors which contribute to vasodilation during shear stress and vascular remodelling [35]. P1 and P2 receptors are also expressed on erythrocytes, platelets, and immune cells [4, 15, 25].

## Roles of purines in the cardiovascular system

### Vascular tone regulation: ATP neurotransmission

It is now accepted that ATP is a vasocontractile cotransmitter in sympathetic nerves in most blood vessels and a vasodilator non-adrenergic non-cholinergic (NANC) cotransmitter in some blood vessels. Geoff had discovered that ATP is an inhibitory neurotransmitter in NANC nerves in gut, and he later called these and other nerves found to utilize ATP as a neurotransmitter “purinergic nerves” [36, 37]. This discovery was important because it provided evidence for physiologically relevant signalling by endogenous extracellular nucleotides. Geoff also proposed that a single nerve may utilize multiple neurotransmitters including ATP; cotransmission [38]. The relevance of these concepts for the cardiovascular system is evident in sympathetic cotransmission of ATP with noradrenaline and neuropeptide Y as shown by Geoff and other researchers in many different blood vessels (see [39, 40]) and for NANC cotransmission of ATP with nitric oxide in rabbit portal vein [41, 42].

The receptors involved in sympathetic neurogenic vasoconstriction to ATP were pharmacologically characterized as P2X receptors [2]. The use of tissue from P2X1 receptor-deficient mice later confirmed an involvement of P2X1 receptors, and by implication ATP, in sympathetic cotransmission [43]. It is now accepted that P2X1 receptors are widely expressed in vascular smooth muscle and mediate excitatory junction potentials and vasoconstriction induced by ATP released during sympathetic cotransmission. There is an increased density of P2X1 receptors at the neuroeffector junction in arterial smooth muscle and cardiac myocytes; they are clustered in lipid rafts and adjacent to sympathetic nerve varicosities from which neurotransmitter is released by exocytosis [44–46]. At high pressure ATP is the predominant sympathetic neurotransmitter in resistance arteries [47], and elevated tone of blood vessels also increases the purinergic component of sympathetic vasoconstriction [48]. The P2X1 receptor rapidly desensitizes which terminates the purinergic vasocontractile response, and receptor sensitivity can be restored following the removal of ATP by its rapid metabolism by ectonucleotidases. Smooth muscle P2Y receptors may be involved in noradrenaline-mediated vasoconstriction involving ATP release via pannexins complexed with α1-adrenoceptors [49].

### Vascular tone regulation: hypoxia, shear stress, and tonic release of purines

Endothelial cells form the innermost layer of blood vessels and, in healthy tissues, provide an ongoing vasodilator tone and inhibit platelet aggregation through the release of factors including nitric oxide, prostacyclin, and hyperpolarising factors. Geoff was interested in the dual control of blood vessel contractility involving perivascular nerves in the adventitia (outermost layer) and endothelial cells adjacent to the vascular lumen. He, with colleagues, showed that ATP is released from endothelial cells and acts as an auto/paracrine signalling molecule at vasodilator endothelial P2Y receptors; the most common are P2Y1 receptors sensitive to ADP and ATP, P2Y2 receptors sensitive to ATP and UTP, and P2Y6 receptors sensitive to UDP. Measurement of physiologically relevant ATP release from cells is challenging because its extracellular concentration is normally kept low (subnanomlar levels) in tissues by rapid degradation by cell surface ectonucleotidases. Another hurdle was that the high intracellular concentration of ATP (mM), which provides a gradient for its release from cells given the appropriate stimuli, meant that high background levels of ATP were an issue due to inadvertent release of ATP by mechanical stimulation and from damaged cells. It sometimes felt that just looking at the preparations caused ATP to pour out. Geoff and colleagues were tenacious and measured an increase in extracellular ATP levels during hypoxia in the heart, concomitant with coronary hypoxic vasodilatation, and also showed that exogenous ATP is a coronary vasodilator [50–52], thus providing evidence that ATP as well as adenosine [53, 54] is a local mediator of hypoxic vasodilatation in the coronary vasculature. Since the in vitro heart preparation was perfused with physiological solution the source of the released ATP was likely the coronary vascular endothelium and there is evidence that hypoxic release of ATP from endothelial cells may be vesicular [55, 56]. Hypoxia also induces ATP release from erythrocytes [57, 58] which can contribute to vasodilatation during hypoxia and ischaemia in vivo*.*

ATP release from endothelial cells is also stimulated by shear stress. The concept of ATP as a local vasodilator during shear stress has gained considerable support since Geoff and colleagues measured an increase in extracellular ATP and UTP levels during an increase in perfusion flow rate in vascular beds and cultured endothelial cell preparations [55, 59–62]. Investigations using mice deficient in endothelial P2Y2 receptors showed that endothelial P2Y2 receptors are involved in flow-induced vasodilatation, eNOS activation, and blood pressure control—the mice developed hypertension [63]. Experiments using P2X4 receptor-deficient mice showed that P2X4 receptors in vascular endothelial cells contribute to vasodilation during shear stress and in vascular remodelling [35]. P2Y1 receptors were shown to be involved in the increase in coronary blood flow following a period of ischaemia in pig hearts implying an auto-/paracrine role for locally released ATP/ADP, possibly from endothelial cells, cardiomyocytes, erythrocytes, and platelets [64].

Shear stress, mechanical deformation, hypoxia, and low pH release ATP from erythrocytes which causes vasodilatation via endothelial P2Y receptors [57, 58, 65–67].

Myogenic tone involves contractile P2Y6 receptors, tonic release of endogenous pyrimidine nucleotides and possibly connexin hemichannels and P2X7 receptors in mouse mesenteric resistance arteries [68]. Myogenic tone of cerebral arterioles is mediated via direct mechanical activation of vasocontractile P2Y4 and P2Y6 receptors rather than the release of endogenous pyrimidine nucleotides [69]. ATP is tonically released from glial cells in rat retinal arterioles in vivo and acts on P2X1 receptors on vascular smooth muscle cells to maintain basal contractile tone [70].

### Cardiovascular diseases, cell damage, inflammation and injury, and trophic signalling

Damage to cells leads to high extracellular levels of nucleotides which can act at smooth muscle vasocontractile P2X1 and P2Y receptors to contribute to vasospasm [15]. ATP can be released from endothelial cells, smooth muscle cells, cardiomyocytes, and erythrocytes by mechanical stimuli including shear and osmotic stress, via mechanisms which include connexin and pannexin channels, which may be relevant in cardiovascular physiology and pathophysiology [71–74]. Nucleotides are released from other cell types including leucocytes and platelets during inflammation and injury [15, 72, 75].

Geoff was interested in purinergic long-term trophic and inflammatory signalling which occurs in vascular remodelling, restenosis, and atherosclerosis and the evidence for dysfunctional purinergic signalling in cardiovascular diseases including hypertension, diabetes, and thrombosis and in heart conditions [15, 25, 27, 76]. Adenosine receptors are involved in angiogenesis through regulating levels of pro- and antiangiogenic factors including VEGF and basic fibroblast growth factor; the process is influenced by the relative expression levels of the four adenosine receptors which can vary depending on the inflammatory conditions [77]. A2A receptors are involved in angiogenesis in the retina and deletion of the receptors reduced neovascularisation in mice [78]. Ectonucleotidases (ENTPDase1 and ecto-5’-nucleotidase) have roles in angiogenesis [79]. P2Y2 receptors are involved in endothelial cell sprouting and blood vessel growth [80, 81]. Tumour hypoxia in cancer activates proangiogenic signalling and generates a chaotic and leaky tumour vasculature which promotes tumour growth and dispersal. Hypoxia in tumours can generate increased extracellular levels of ATP within tumours [82]. Roles for P2X7 and P2Y11 receptors have been described in ATP-mediated inhibition of tumour endothelial cell migration [83].

Deletion of P2Y2 receptors on endothelial cells promotes atherosclerotic plaque stability [84]. P2X1 receptor activation can inhibit smooth muscle cell proliferation [85]. Individuals with type 2 diabetes have reduced tissue perfusion, lower plasma ATP concentrations and lower blood flow during exercise and hypoxia and impaired endothelium-dependent vasodilatation compared with controls [86]. Release of ATP from cells by mechanical stimulation (including shear stress, osmotic pressure, strain and compression) is elevated in inflammation and injury and is attenuated in hereditary and metabolic conditions including erythrocytes in cystic fibrosis [74].

Perivascular adipose tissue (PVAT), a layer of fatty tissue which surrounds blood vessels, is now known to release a variety of adipokines and other factors which regulate vascular tone [87, 88]. Adipose tissue expresses P2 receptors which can be activated by ATP released from perivascular sympathetic nerves to regulate lipolysis [89–91], and there is evidence for constitutive release of nucleotides from adipocytes in regulation of lipolysis [92]. In obesity, the increase in adipocyte area and mass leads to hypoxia [88]. Since hypoxia stimulates purine release from cells, the role of purinergic signalling in PVAT in vascular tone regulation and remodelling requires further investigation.

Platelets express P2Y1, P2Y12, and P2X1 receptors [93]. The P2Y12 receptor antagonists clopidogrel, prasugrel, and tricagrelor are used in the treatment of thrombosis, stroke, and myocardial infarctions in patients. Purine receptors, ectonucleotidases, and purinergic release and uptake mechanisms are potential therapeutic targets in cardiovascular disease.

## Memories of Geoff’s laboratory at UCL (~1985—1997)

Geoff was a positive force at the Department of Anatomy, UCL where he and his team of dedicated postdocs and PhD students made discoveries on the expression and roles of purine receptors in the cardiovascular and other systems. Geoff understood the need for collaboration and translational research in science—his office was a hub for meetings with clinicians and leaders in the field—and the importance of being abreast of new scientific research techniques, which he achieved by bringing the expertise to his laboratory or through external collaborations. Geoff travelled frequently to conferences throughout the world where he appeared to adopt scientists—each trip seemed to be followed by the addition of a new visiting scientist or PhD student to the group. This led to a very eclectic, multicultural, and multinational team which contributed to the group’s vibrancy and promoted ideas and collaborations. The camaraderie was great. Geoff supported and looked after his team. There were frequent seminars with, importantly, post-seminar food and wine to facilitate discussions. We had fun. Geoff recognized the importance of young scientists and encouraged them. A student’s first accepted paper or successful completion of their PhD was celebrated (more food and wine) and Geoff would seek out the student to give them his warm congratulations.

Individual research meetings were held monthly in Geoff’s magnificent high-ceilinged office at UCL. Banks of filing cabinets lined the walls and held thousands of research articles (photocopying journal articles in the library in the days before the internet was a way for PhD students to earn extra funds); Geoff knew exactly the location of each publication and its content and would pull out articles to illustrate a point. Typically Geoff would chat about news about himself or others before discussing lab results and his openness fostered trust and engagement. He made each one of us feel valued. Geoff jotted down ideas in handwriting that was as exuberant as he was but not always easy to read. He expected full commitment which he generally achieved through his enthusiasm, praise, and encouragement. At one research meeting I mentioned an interest in visiting Australia and Geoff later facilitated this by encouraging me to apply for a Royal Society Travel Fellowship and identified colleagues of his in Melbourne as potential hosts. I thank him also for his support in my application to the Royal Society for a University Research Fellowship, which led to us collaborating with Professor Mike Spyer to investigate purinergic signalling in central nervous system control of the cardiovascular system at the Royal Free Hospital School of Medicine, London [94, 95]. Geoff was a great mentor.

## Conclusion

**Fig. 1:**
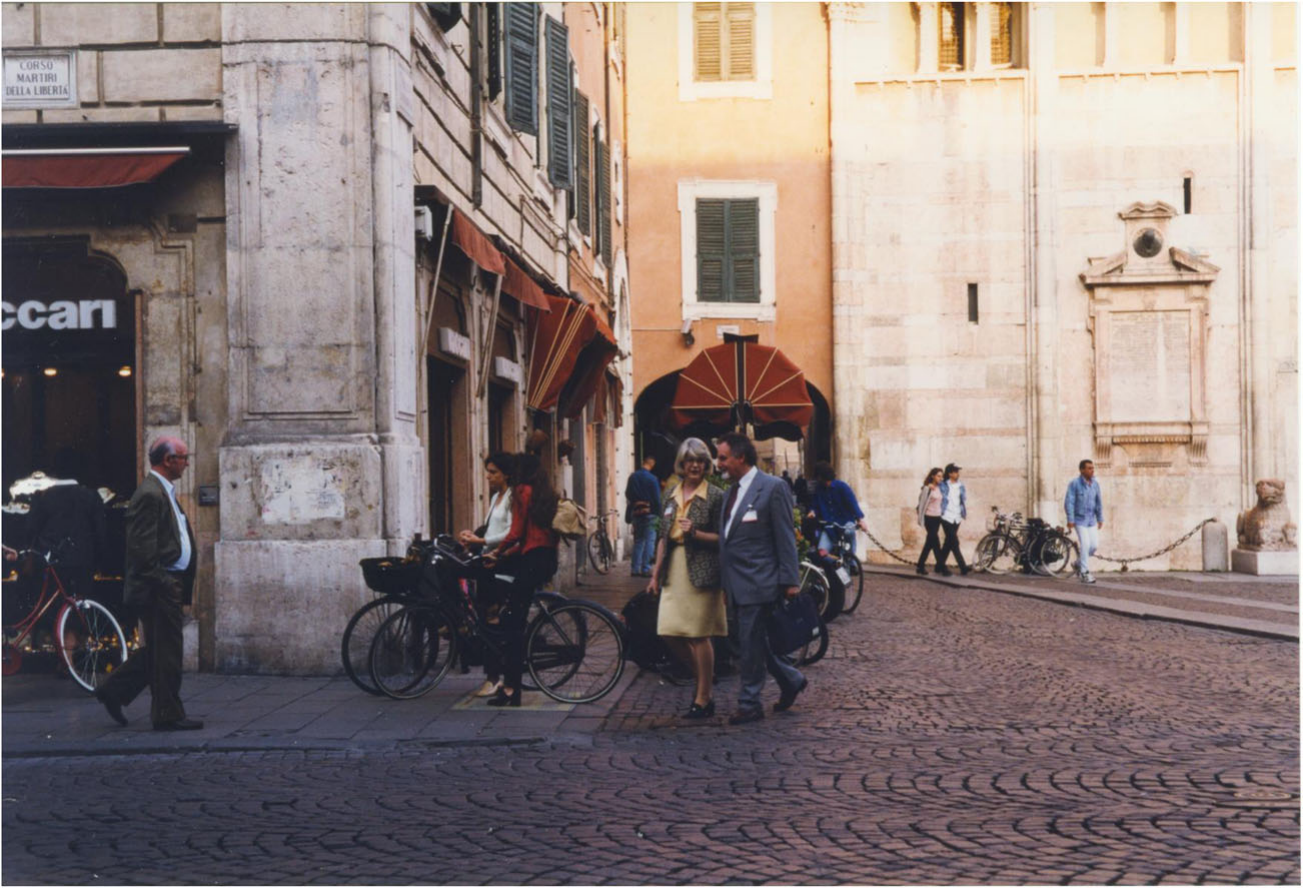
Geoff Burnstock with Maria Teresa Miras-Portugal at the 6th International Symposium on Adenosine and Adenine Nucleotides, Ferrara, Italy, May 1998

Geoff championed the role of ATP as an extracellular signalling molecule despite opposition to the concept that ATP was other than an intracellular energy source, and he was fearless and unshakeable in his conviction [39, 40]. Our understanding of purinergic signalling in the cardiovascular system has been shaped by Geoff Burnstock through his groundbreaking discoveries. Geoff thrived on interactions with the international scientific community, and he promoted communication and collaboration through his initiation of the Purine Clubs and his journal *Purinergic Signalling*. A photograph of Geoff which I took at the 6th International Symposium on Adenosine and Adenine Nucleotide in Ferrara, Italy (1998) (Figure 1) is one of my favourites and is how I like to remember Geoff—animated, engrossed in conversation (here with Maria Teresa Miras -Portugal) and always chatting about purines. Geoff shone. Geoff lives on through his enormous scientific contribution and in our memories.
